# Oxidative Stress in Parkinson's Disease: Potential Benefits of Antioxidant Supplementation

**DOI:** 10.1155/2020/2360872

**Published:** 2020-10-12

**Authors:** Sandro Percário, Aline da Silva Barbosa, Everton Luiz Pompeu Varela, Antônio Rafael Quadros Gomes, Michelli Erica Souza Ferreira, Thayana de Nazaré Araújo Moreira, Maria Fani Dolabela

**Affiliations:** ^1^Oxidative Stress Research Laboratory, Institute of Biological Sciences, Federal University of Pará, Av. Augusto Corrêa, 01, Belém, Pará, Brazil 66075-110; ^2^Institute of Health Sciences, Federal University of Pará, Av. Augusto Corrêa, 01, Belém, Pará, Brazil 66075-110

## Abstract

Parkinson's disease (PD) occurs in approximately 1% of the population over 65 years of age and has become increasingly more common with advances in age. The number of individuals older than 60 years has been increasing in modern societies, as well as life expectancy in developing countries; therefore, PD may pose an impact on the economic, social, and health structures of these countries. Oxidative stress is highlighted as an important factor in the genesis of PD, involving several enzymes and signaling molecules in the underlying mechanisms of the disease. This review presents updated data on the involvement of oxidative stress in the disease, as well as the use of antioxidant supplements in its therapy.

## 1. Introduction

Parkinson's disease (PD) is considered cosmopolitan and makes no distinction between social classes or between races, affecting both men and women, especially in the age range between 55 and 65 years, but it tends to occur with greater frequency in men [1, 2].

It is estimated that this disorder affects approximately 1% of the world population older than 65 years, representing up to 2/3 of all patients with movement disorders throughout the world [3]. PD has become increasingly more common with advances in age, reaching proportions of 2.6% of the population over 85 years old.

According to Silberman et al. [4], the number of individuals older than 60 years has been increasing, as has life expectancy in developing countries. Thus, along with health issues associated with an aging population, PD also imposes a significant impact on the economic, social, and health structures of these countries [5]. Therefore, a greater knowledge about the disease and an improvement of the planning of public health to minimize its impact in the future are necessary. Moreover, it is estimated that by 2020, approximately 40 million people worldwide will develop motor disorders secondary to PD [2, 6].

## 2. The Involvement of Oxidative Stress in PD

Oxidative stress is the result of many metabolic processes essential to the body. On the other hand, it can exert a toxic and deleterious role in the body [7–8].

**Figure 1:**
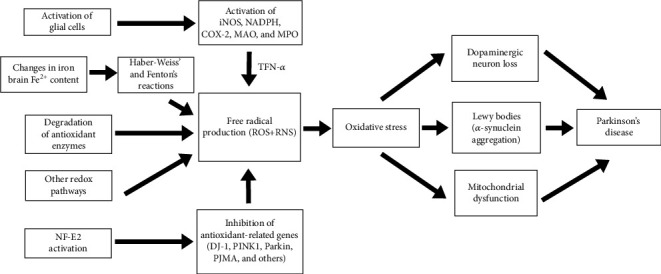
Sources of oxidative stress in Parkinson's disease.

Oxidative changes are highlighted as an important factor in the genesis of Parkinson's disease (Figure 1), with the activation of glial cells being the main source of oxidative stress [9]. Some key enzymes are involved in the genesis of oxidative species derived from oxygen and nitrogen, namely, reduced nicotinamide adenine dinucleotide phosphate oxidase (NADPH), inducible nitric oxide synthase (iNOS), and astrocytic myeloperoxidase (MPO) [10–16], as well as inflammatory factors, such as tumor necrosis factor alpha (TNF-*α*) [17] and cyclooxygenase-2 (COX-2) [9].

During the pathogenesis of PD, the production of oxygen-reactive species damages the *substantia nigra* through lipid peroxidation, protein oxidation, and DNA oxidation. This phenomenon seems to be induced mainly by changes in iron content of the brain, mitochondrial dysfunction, monoamine oxidase (MAO) activation, or even by changes in the antioxidant defense system [18–23].

Additional redox pathways involved in PD are androgen receptor-induced neurodegeneration [24], production of oxidatively modified forms of *α*-synuclein and increased *α*-synuclein aggregation [25, 26], degradation of antioxidant enzyme quinone oxidoreductase 1 (NQO1) [27], reduction of the deglycase activity of protein DJ-1 [28], activation of gene LRRK2 [29], and tetrahydrobiopterin (BH4) and tyrosine hydroxylase (TH) metabolism impairment [30].

Other evidence of oxidative stress involvement in PD was given by Colamartino et al. [31], who demonstrated that L-dihydroxyphenylalanine (L-DOPA) therapy decreases markers of lipid and protein peroxidation and increases total levels of reduced glutathione (GSH). In addition, L-DOPA and carbidopa can reduce damage to DNA and micronuclei induced by hydrogen peroxide (H_2_O_2_) *in vitro*.

Indeed, increased levels of oxidative stress markers are already found in blood from PD patients [32–34] and animal models of the disease [35].

In this sense, Farias et al. [36], investigating the peripheral biomarkers of reactive oxygen species (ROS) and reactive nitrogen species (RNS) in PD patients, found increased lipid hydroperoxides (LOOH), malondialdehyde (MDA) levels, and superoxide dismutase (SOD) activity, alongside decreased catalase (CAT) activity. Furthermore, these authors suggest that MDA may be a PD biomarker, while LOOH and SOD would be associated with late PD features.

To study oxidative changes in this neurodegenerative disease, an experimental mouse model of the disease is often used, in which damage to the dopaminergic neurons of the *substantia nigra pars compacta* is induced by the administration of 1-methyl-4-phenyl-1,2,3,6-tetrahydropyridine (MPTP), which promotes activation of microglial cells [9]. The peripheral administration of this neurotoxin promotes important gliosis, accompanied by increased activation of iNOS in the *substantia nigra*, as well as of NADPH oxidase and MPO.

Furthermore, protein oxidation has already been identified as a marker of oxidative damage in *postmortem* brain tissue from PD patients [37].

Abraham et al. [38] evaluated the possibility of oxidative damage to red blood cells of PD patients by evaluating the activity of antioxidant enzymes, verifying that the activities of SOD, CAT, glutathione peroxidase (GSH-Px), and glucose-6-phosphate dehydrogenase (G6PD) were significantly lower in PD patients. Consequently, these authors suggested the involvement of oxidative stress as a risk factor for the disease and pointed out its importance in the underlying mechanisms of neurodegeneration in PD.

Venkateshappa et al. [39] evaluated the redox state of the *substantia nigra* and *caudate nucleus* during physiological aging in the human brain by assessing the expression of glial fibrillary acidic protein (GFAP) and activity of mitochondrial complex 1. The authors observed a significant increase in protein oxidation, loss of mitochondrial complex 1 activity, and increase in astrocytic proliferation in the *substantia nigra* compared to the *caudate nucleus* as age increased. These changes in the *substantia nigra* were attributed to a significant decrease in the antioxidant function represented by SOD, GSH-Px, and GSH, and a decreasing trend of GSH and CAT with age. However, these parameters showed no significant differences in the *caudate nucleus*. These results led the authors to suggest that the *substantia nigra* suffers extensive oxidative damage, loss of antioxidants and mitochondrial function, and increased expression of GFAP during physiological aging, changes that could make it more vulnerable to neurotoxic environments, thereby contributing to selective degeneration during the evolution of PD.

**Figure 2:**
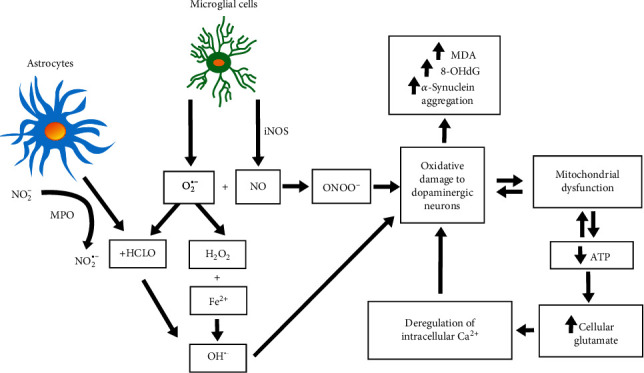
Mechanisms of free radical involvement in Parkinson's disease.

The oxidative imbalance involved in PD neurodegenerative processes seems to be a multifactorial phenomenon triggered by factors such as the aging of the brain, genetic predisposition, mitochondrial dysfunction, production of free radicals, and environmental toxins [15, 35, 40–41]. Nevertheless, some mechanisms are of key importance for the development of PD (Figure 2).

### 2.1. Iron and Iron-Dependent Free Radical Production

Iron also plays an important role in the oxidative changes of Parkinson's disease, as it is present in various regions of the brain, noteworthy in dopaminergic neurons of the *substantia nigra* [42–44]. Iron accumulation associated with neuromelanine can represent one of the causes of increased free radicals in the *substantia nigra* and consequently lead to oxidative stress and neurodegeneration [42, 45].

Being rich in this metal, dopaminergic neurons are very susceptible to Fenton's or Haber-Weiss' reactions, which convert H_2_O_2_ to hydroxyl radicals, powerful oxidizing agents. Therefore, the presence of large quantities of ferrous ions (Fe^2+^) in the *substantia nigra* promotes high oxidative stress, DNA damage, and cell death by autophagy [46]. Additionally, under the action of SOD, free radical superoxide (O_2_^•-^) undergoes dismutation to H_2_O_2_, which, in the presence of high concentrations of iron, produces hydroxyl radicals (OH^•^) through Fenton's reaction.

In this context, Hochstrasser et al. [47] and Olivieri et al. [48] studied the role of ceruloplasmin in this disease, an extracellular ferroxidase that oxidizes iron from its toxic ferrous form to the nontoxic ferric form. Analyzing the cerebrospinal fluid of patients with PD, Olivieri et al. found higher levels of ceruloplasmin oxidation in these patients than in controls or in subjects with other neurodegenerative diseases. Similarly, ceruloplasmin-deficient mice showed accumulation of iron in the central nervous system and increased lipid peroxidation [49], and ceruloplasmin deficiency due to copper dyshomeostasis is reported in PD patients [50]. Treatment with another iron chelator, lactoferrin, also offered protection against oxidative stress in MPTP-induced PD mice [51].

Moreover, iron can lead to the formation of Lewy bodies through the aggregation of *α*-synuclein [52–57]. Alpha-synuclein is an abundant protein in presynaptic terminals and is responsible for the formation of Lewy bodies—mainly by its iron-dependent binding to cytochrome *c* and mitochondrial damage—via regulation of mitochondrial complex 1, increasing susceptibility of the *substantia nigra* to free radicals in PD [58–63]. Bayir et al. [61] and Rostovtseva et al. [64] investigated the biochemical mechanism of action of *α*-synuclein and showed that this protein can bind to anionic lipids (such as cardiolipin), exerting peroxidase function while protecting *nigral* neurons against damage by H_2_O_2_ and consequently preventing apoptosis. Shahnawaz et al. [65] suggest that detection of *α*-synuclein by protein misfolding cyclic amplification in cerebrospinal fluid may provide an efficient biochemical test for the diagnosis of PD.

Reinforcing the importance of iron in the underlying neuropathogenic changes of PD, the use of iron chelators in models of nigral neurodegeneration induced by proteasome inhibitors showed a decreased loss of dopaminergic neurons as well as a decreased *α*-synuclein aggregation and a consequential reduced formation of Lewy bodies [54, 66].

In addition, the toxic effects of oxidative stress seem to be boosted by environment-present substances, such as herbicide paraquat (1,1′-dimethyl-4,4′-bipiridina dichloride), frequently used in agriculture, which operates in synergism with iron when absorbed by the organism. As a consequence of paraquat poisoning, the increase in free radical production in several areas of the body, including the *substantia nigra*, peters out the antioxidant capacity of SOD and CAT, and promotes cell death [20, 67].

### 2.2. Mitochondrial Dysfunction

Mitochondrial dysfunction is commonly associated with neurodegenerative diseases. In PD, genetic mutations associated with the mitochondria and the action of toxic agents, such as rotenone and MPTP, lead to failures in the electron transport chain and the consequent increase in oxidative stress, accumulation of intracellular Ca^2+^, glutamate excitotoxicity, and decrease in energy production, culminating in neuronal damage and death [68–69].

Deficiency of complex 1 (NADH-ubiquinone oxidoreductase), a macrocomplex in the electron transport chain encoded by mitochondrial DNA, seems to be one of the causes of oxidative stress increase and bioenergetic deficiency in PD [70–72]. However, the mechanisms by which it occurs in PD are not fully elucidated. Nevertheless, it is known that the oxidation of cysteine residues by iron culminates in mitochondrial dysfunction in experimental models of the disease [73].

Studies that verify the toxic effects of rotenone on complex 1 demonstrate that its partial inhibition is related to increased levels of superoxide radicals. In addition, oxidative stress potentiates the deregulation of intracellular Ca^2+^ induced by the accumulation of glutamate, leading to cell death by necrosis. Moreover, the accumulation of intracellular glutamate increases the demand for ATP, diminishing mitochondrial respiratory capacity and causing a failure in the electron transport chain [68, 74–75].

Alternatively, the decrease in the activity of complex 1 may be related to mechanisms of intracellular self-oxidation due to mitochondrial abnormalities or failures in complex 1 assembly [60, 76].

Indeed, the association between mitochondrial dysfunction and oxidative stress seems perfectly relevant in PD, since mitochondrial heat-shock proteins, such as mortalin, mitochondrial heat-shock protein 70 (mtHsp70), and glucose-regulated protein 75 (GRP75), were found to be significantly increased in patients with the disease [77–79].

### 2.3. Oxidative Stress-Mediated Gene Expression

Some authors attribute the regulation of PD genes to oxidative stress. Among them, the DJ-1 gene appears to be a preponderant factor for the development of PD. When active, the gene decreases the expression of oxidative stress markers and prevents neurological damage. The opposite occurs when this gene is inactivated or mutated: markers of stress increase, as well as the predisposition to disease [80–88]. Such a mechanism seems to be related to residues of cysteine inherent to the DJ-1 gene [89]. In addition, this gene induces the synthesis of glutathione and inhibits the toxicity of *α*-synuclein [28, 90].

Steckley et al. [91], Qi et al. [92], and Feng et al. [93] attributed the regulation of oxidative stress and, consequently, neuronal apoptosis to the gene PUMA, one of the genes of the Bcl-2 family. This gene is responsible for the permeability of the mitochondrial membrane.

Activation of the LRRK2 gene may also be responsible for increased oxidative stress and neuronal loss in PD [29].

As suggested by Chen et al. [94] and Haskew-Layton et al. [95], the negative regulation of genes related to antioxidant defenses via stimulation of nuclear factor erythroid 2 (NF-E2) is positively correlated with the destruction of astrocytes. The same happens with PTEN-induced putative kinase 1 (PINK1), a gene that inhibits mitochondrial dysfunction and is positively related to neuroprotection [96].

According to Cook et al. [97], mutations in the *parkin* gene and the abnormal accumulation of *α*-synuclein proteins in certain dopaminergic neurons are closely related to PD and oxidative stress. In this sense, Basso et al. [98], by inhibiting transglutaminase, observed a reduction in markers of oxidative stress and a decrease in neuronal death, features of PD.

### 2.4. Role of Nitric Oxide (NO)

Some studies suggest that NO plays an important role as a mediator of the neurotoxicity associated with mitochondrial damage in several neurological disorders, such as PD [99]. Under pathological conditions, the expression of iNOS and NADPH oxidase activity occurs in microglia, leading to high production of NO and O_2_^•-^. These two free radicals react to produce peroxynitrite radicals (ONOO^−^), a highly reactive molecule that can cause damage to dopaminergic neurons.

Evidencing the importance of NO synthesis and its byproducts in the physiopathology of PD, nitration of tyrosine residues is a known marker of oxidative stress in patients with Parkinson's disease and is induced by ONOO^−^ [100–101]. In this context, Sue et al. [102] studied the effects of ethyl pyruvate (EP), a known scavenger of reactive oxygen species, in mice treated with MPTP, demonstrating that EP mitigates iNOS expression in the *substantia nigra*, reducing oxidative damage.

Similarly, Yeung et al. [103] demonstrated that aldose reductase deficiency, a tyrosine hydroxylase cofactor involved in dopamine synthesis, can induce oxidative stress by increasing NO and nitrite (NO_2_^−^), causing the loss of dopaminergic neurons and autophagic abnormalities in animals with PD.

Notwithstanding, iNOS knockout mice are more resistant to the neurodegenerative effects of MPTP than wild-type mice [10]. The same effect is observed in animals treated with specific inhibitors of neuronal nitric oxide synthase (nNOS), such as 7-nitroindazole [14].

Conversely, Rathnayake et al. [17] identified that low serum NO metabolites (nitrites and nitrates (NOx)) are associated with cognitive impairment in PD patients, proposing NOx as a marker of early-stage PD.

Indeed, the dopaminergic neurotoxin MPTP is associated with the induction of iNOS in the *substantia nigra*, leading to the formation of ONOO^−^ [52, 104], and the administration of MPTP in rats induces considerable gliosis in the *substantia nigra*, as well as a significant positive regulation over iNOS [105]. Moreover, in iNOS gene-deficient rats, the neurodegenerative effects of MPTP administration were less prominent, suggesting the inhibition of iNOS as a potential target for drugs in the treatment of PD [105]. Additionally, the use of nitric oxide synthase (NOS) inhibitors prevents dyskinesia in Parkinson's disease, at least in part via inhibition of glial cell activation and iNOS expression, showing the role of NO in the pathogenesis of PD [106]. Moreover, astrocytes express high levels of MPO, which produce hypochlorous acid (HOCl) from the reaction of H_2_O_2_ and chloride ions (Cl^−^), causing additional oxidative damage. The presence of HOCl can increase the amount of OH^•^, as HOCl can also react with O_2_^•-^. Myeloperoxidase also catalyzes the conversion of nitrite from its nonreactive form (NO_2_^−^) to its free radical form (NO_2_^•-^), enhancing protein damage [9].

This was also evidenced in the experimental model of neurodegeneration proposed by Ebadi and Sharma [107], in which the activation of iNOS, the synthesis of NO, and the generation of peroxynitrite were associated with *nigrostriatal* dopaminergic neurodegeneration and that animals that overexpressed the genes metallothioneins 1 and 2 showed greater protection against damage caused by oxidative stress due to iNOS activation.

### 2.5. Role of MAO, MPO, and NADPH Oxidase

In addition to the free radical-generating processes already mentioned, the reactions catalyzed by MAO are also potential free radical generators and are related to the decrease in intrinsic antioxidant defenses [108–109].

At the intracellular level, dopamine is degraded both by MAO and by autooxidation [110–111]. The metabolism of dopamine leads to the formation of dihydroxyphenylacetic acid (DOPAC) and H_2_O_2_ [112]. The autooxidation of intracellular dopamine produces H_2_O_2_ and dopamine-quinone, which participate in nucleophilic reactions associated with sulfhydryl groups, leading to further reduction of GSH-Px activity [113].

Dopamine-quinone is also capable of inhibiting the function of the dopamine transporter within synaptosomes by inhibiting the enzyme tyrosine hydroxylase, resulting in incomplete ATP synthesis [114–116]. The ratio between GSH and oxidized glutathione (GSSG) is decreased during synaptosome degeneration, thus propitiating the formation of even more free radicals [117–118]. Furthermore, the decrease in the GSH/GSSG ratio can impair free radical scavenging by GSH, as a reflection of constant oxidation of the GSH molecule and consequent depletion of cellular GSH [119]. In studies with cells in culture, GSH depletion has been related to the toxicity of dopamine and H_2_O_2_ [120].

In addition to dopamine metabolism, MAO can metabolize MPTP by the action of MAO-B. MPTP is oxidized to dihydropyridine (MPDP^+^) and converted to N-methyl-4-phenylpyridine (MPP^+^) by autooxidation, binding to dopamine transporter proteins. Subsequently, it is retaken by dopaminergic *nigral* neurons [121]. Once in the cytosol, MPP^+^ promotes the inhibition of complex 1, as well as the production of free radicals (through the activation of NADPH oxidase, microglial iNOS, and astroglial myeloperoxidase) and the production of proinflammatory cytokines, such as TNF-*α* and interleukin-1*β* (IL-1*β*). These phenomena contribute to the death of dopaminergic neurons in experimental models of PD [12–13, 122].

Likewise, MPO is an important component of the PD puzzle. In *postmortem* mesencephalic analysis of PD patients, Choi et al. [13] observed significantly higher levels of MPO than in controls. In the same study, using the MPTP model of PD, they found high levels of 3-chlorotyrosine, a marker of MPO protein damage. These authors also demonstrated that MPO-deficient mice are resistant to MPTP neurotoxicity. In parallel, Maki et al. [16] also demonstrated that MPO plays an important role in oxidative damage to *α*-synuclein. Moreover, the prooxidant effect of MPTP in animal models of PD was minimized using paroxetine (an antidepressant drug), which promoted the reduction of astroglial MPO expression, production of ROS through NADPH oxidase, and the expression of proinflammatory cytokines, decreasing the loss of dopaminergic neurons and improving motor functions. These effects suggest the role of oxidative stress in the pathogenesis of PD, and therefore, the use of drugs designed to decrease the neurodegenerative effects caused by free radicals displays great potential for the treatment of the disease [123].

Furthermore, the role of NADPH oxidase in oxidative damage in PD was demonstrated through the treatment of PD-induced mice with a nonselective agonist of cannabinoid receptor. This treatment promoted suppression of O_2_^•-^ production by NADPH oxidase in the *microglia*, and oxidative damage to nucleic acid and protein levels were reduced [124]. The damage to nucleic acid was evaluated by the dosage of 8-hydroxy-2-deoxyguanosine (8-OHdG), a marker of oxidative damage to the DNA. Likewise, 8-OHdG was elevated in the cerebrospinal fluid of patients with PD in comparison to control subjects [32].

## 3. Antioxidant Approaches to PD

Considering all factors related to oxidative stress overstimulation in the underlying mechanisms of PD, numerous studies have suggested the potential beneficial effects of antioxidant supplementation in PD treatment, and several approaches have been attempted so far, from traditional antioxidant schemes, such as vitamin E, C, and *β*-carotene supplementation, to more innovative and bold approaches, such as the use of nanoparticles to deliver antioxidant molecules, among several others.

Indeed, several studies show that brains from PD patients present low levels of endogenous antioxidants, such as glutathione and coenzyme Q_10_ (CoQ_10_) [125], increased oxidation of dopamine [115], and high levels of iron [126], suggesting that oxidative stress plays a crucial role in the pathology of PD. Considering the greater iron content of some areas of the brain [127], low levels of GSH are expected [128], as well as increased lipid peroxidation [129] and oxidation of nucleic acids [130].

In addition, Campolo et al. [131] suggest that the reduction of the total antioxidant capacity observed in the PD prodromal, and when associated with olfactory loss and cardiovascular dysautonomia, may represent a useful biomarker for an early and integrative PD diagnosis.

In this sense, antioxidants can provide a significant advance in the therapeutic treatment of PD, as it is believed that Parkinson's neurodegeneration is linked to dietary habits and that nutritional deficiency of antioxidant compounds, such as folic acid [132], vitamins (A, C, E, and niacin), and selenium, increases the risk of subjects developing PD [133–134]. Thus, the therapeutic approach for the treatment of PD must include the modulation of oxidative stress using antioxidants, which, at least partially, may be provided by an adequate diet.

Several antioxidant molecules have been used both in experimental and clinical studies of PD and will be categorized and presented henceforth by its source or chemical class when appropriate.

### 3.1. Endogenous Molecules

#### 3.1.1. Melatonin

A natural antioxidant capable of reducing cellular oxidative stress, melatonin protects mitochondrial functions *in vitro*. Low levels of melatonin were found in PD patients [135]. Zampol and Barros [136] prompted a study indicating that melatonin administration to cultured cells reversed *α*-synuclein damage to mitochondria. Additionally, Patki and Lau [137] investigated whether melatonin could reverse neurobehavioral deficits and mitochondrial disorders in an experimental model of PD, suggesting that, in the long term, melatonin protects not only mitochondria but also neurons in an animal model of chronic PD. Due to this factor, melatonin can potentially be effective in slowing the progression of idiopathic Parkinson's disease and reducing oxidative stress and respiratory chain inhibition in other mitochondrial diseases. In a similar study, Paul et al. [138] identified that the administration of melatonin protects against behavioral deficits and loss of nigral dopamine and reduces oxidative stress by eliminating OH^•^ radicals and boosting the activity of antioxidant enzymes in an animal model of PD. Similar results were observed by Li et al. [139] and Rasheed et al. [140]. Curiously, despite promoting the reversion of several rotenone- [141] and 6-OHDA-induced damage in rats [142], melatonin supplementation to animals was unable to improve locomotor activity. In addition, administration of melatonin to humans promoted reduction of COX-2 activity, nitrites and nitrates, and lipid peroxides that correlated with clinical improvement of PD patients [143]. Nevertheless, the association of melatonin with L-DOPA significantly decreased the side effects of L-DOPA therapy in mice [144]. A particular aspect of melatonin administration in PD lies on its effect on the occurrence of sleep disorders, a common finding in PD patients. In this regard, melatonin treatment promoted sleep improvement in animal studies [145–146], while its effect on clinical trials is controversial [147–149]. Notwithstanding, one meta-analysis study suggests melatonin therapy as highly indicated for the treatment of sleep disorders in PD patients [150].

#### 3.1.2. Coenzyme Q

Another important antioxidant system is represented by CoQ_10_, a mitochondrial electron carrier that also acts in the prevention of oxidative damage [151–152]. It also acts as a cofactor and activator of proteins of mitochondrial coupling [153]. However, the mechanisms by which CoQ_10_ protects dopaminergic neurons against degeneration are still not well understood, although it is known that the reduction of CoQ_10_ levels in PD patients induces changes in ATP synthesis and damage to the mitochondrial membrane [125]. In this sense, oral administration of CoQ_10_ in animal models and in patients with PD caused a continuous decrease in mitochondrial dysfunction [154–155], loss of dopamine and dopaminergic axons [156], protection of dopaminergic neurons against excitotoxin-induced neurodegeneration in PD [157–158], and partial improvement of motor performance [159]. However, a clinical trial conducted with 600 patients showed no evidence of benefit for CoQ_10_ supplementation [160], a result supported by a recent meta-analysis [161].

#### 3.1.3. Urate

High levels of urate have been associated with a lower risk for PD [162], and changes in urate levels can predict the development of PD in animal models of the disease [163]. Coolen et al. [164], in a study with daily oral supplementation of 5000 mg of ATP in humans, identified that there was an increase in uric acid. In parallel, Andreadou et al. [165] detected the presence of reduced serum levels of this antioxidant molecule in patients with PD and suggested the potential use of this molecule in the therapy of the disease. Indeed, feeding a 1% uric acid diet to rats reversed PD symptoms [166], effects that may be related to NF-E2-related factor 2 (Nrf2) bound to the antioxidant response element (Nrf2-ARE) pathway [167]. Moreover, administration of inosine, a urate precursor, was safe and promoted improvement of PD symptoms in humans [168].

#### 3.1.4. *β*-Nicotinamide Adenine Dinucleotide (NAD)

NAD is known to decrease in PD [169]. To investigate whether NAD replenishment is beneficial in a 6-OHDA-induced mouse model of PD, Shan et al. [170] injected NAD in the striatum, resulting in less motor deficits and dopaminergic neuronal damage to the animals.

#### 3.1.5. Kynurenic Acid (KA)

KA and quinolinic acid (QA) are metabolites of tryptophan degradation and have important neurological activities. KA/QA ratio changes are associated with neurological disorders, such as PD. KA administration prevented QA-induced brain damage in an *ex vivo* rat model of PD, preventing changes in Nrf2 levels, oxidative damage, and mitochondrial dysfunction [171].

#### 3.1.6. L-Carnitine

Reactive gliosis and neuroinflammation are features of PD and might result from fatty acid oxidation. In this sense, L-carnitine inhibited lipopolysaccharide-induced oxidative stress in microglial cells, reversing the effects of detrimental neuroinflammation in vitro [172].

#### 3.1.7. Glutamine

Glutamine has a positive role in reducing oxidative stress damage and suppressing MPTP-induced cytotoxicity in cultured PC12 cells. Moreover, glutamine restores SOD, GSH-Px, and lipid peroxidation markers to basal levels in those cells, probably through inhibition of the PI3K/Akt signaling pathway [173].

#### 3.1.8. *n*-3 Polyunsaturated Fatty Acids

Omega-3-polyunsaturated fatty acids (*n*-3 PUFA) have been widely associated with beneficial effects over different neurodegenerative diseases, such as PD. Hernando et al. [174] tested the effects of docosahexanoic acid (DHA) and its hydroxylated derivative, DHAH, in a 6-OHDA-induced animal model of PD, showing a positive effect on Nrf2 pathway regulation in the treated group due to the potential antioxidant effect of these compounds.

#### 3.1.9. Sulfur-Containing Antioxidants

Among the endogenous antioxidant molecules, some can easily promote a reducing environment within the cytoplasm, due to the particular aspects of the interaction between the intracellular environment and sulfur-hydrogen bonds that are present in these molecules. This provide these molecules with special antioxidant properties; thus, they are discussed as a separate group, alongside with N-acetylcysteine, an exogenous molecule, yet an important precursor of endogenous GSH synthesis.

*(1) Lipoic Acid (LA)*. Lipoic acid is another potent antioxidant that promotes the removal of free radicals and increases antioxidant defenses, boosting the levels of GSH, *α*-tocopherol, and ascorbic acid. LA can promote the reduction and prevention of oxidative stress, either in its oxidized form (LA) or in its reduced form, dihydrolipoic acid (DHLA). Due to this ability to prevent neuronal damage caused by ROS in the nervous system, LA supplementation has been suggested for the therapy of neurodegenerative diseases, including PD [175–177]. Bilska et al. [178] demonstrated that LA administration enhances the antioxidant defense system, slows the progression of neuronal degeneration, and improves the regeneration of injured tissues. This may be due to the increase in both GSH levels and activity of GSH-Px and glutathione-S-transferase (GST). Likewise, Zhou et al. [179] demonstrated that administration of alpha lipoamide, a neutral amide derivative of alpha-lipoic acid, restored the number of dopaminergic neurons in the midbrain and recovered mitochondrial function in an animal model of PD. Zhou and Cheng [180], in a 6-hydroxydopamine- (6-OHDA-) induced model of PD, demonstrated that LA alleviated 6-OHDA-induced cell injury, possibly by inhibiting autophagy mediated by the AMPK/mTOR pathway. These neuroprotective effects of lipoic acid were also observed for a combination of carnosine–alpha-lipoic acid in a model of early-stage PD [181]. Moreover, Zhang et al. [182] demonstrated that LA alleviates L-DOPA-induced dyskinesia in rats, and similar results were presented by Abdin and Sarhan [183], who found normalization of catalepsy score and apparent preservation of striatal integrity in rotenone-induced PD in rats.

*(2) Reduced Glutathione*. Among the endogenous antioxidant systems, the main antioxidant system seems to be the redox system of GSH, which protects cells against oxidative stress through three different pathways: direct scavenging of reactive oxygen species, transition metal chelation, and antioxidant cofactors (GSH is required for GSH-Px activity). The essential elements of these systems are GSH-Px, which reduces hydrogen peroxide or lipid peroxide, and GST, which combines electrons to GSH and some ATPase and, therefore, may reduce GSSG or GSH conjugates [184–185]. According to Yamamoto et al. [186], the inhibition of proteasomes induces GSH synthesis to protect nerve cells from oxidative damage. On the other hand, the decrease in glutathione levels results in oxidative stress and mitochondrial dysfunction, regarded as triggering factors in PD neurodegeneration [187]. It is also believed that a reduced GSH/GSSG ratio can increase ROS and RNS production [118] through the opening of GSH redox state-dependent transition pores of mitochondrial permeability [188]. Furthermore, high levels of ROS and RNS may also impair the operation of complex 1 by oxidation of significant residues within the complex and consequent reduction of glutathione reductase (GR) activity, an enzyme responsible for the reduction of GSSG [189–190]. Despite the inability of GSH to cross the blood-brain barrier [191], Sechi et al. [192] administered GSH intravenously to untreated PD patients and found significant improvement for all subjects. Alternatively, glutathione analogs were also employed. Yamamoto et al. [193] tested YM737—a GSH analog—in a rat model of PD, with better results than GSH itself. Wassef et al. [194], who performed studies in transgenic *Drosophila melanogaster* flies overexpressing *α*-synuclein and methionine sulfoxide reductase (MSRA), observed that dietary supplementation with S-methyl-L-cysteine was able to prevent or alleviate the symptoms of PD since it participates in the antioxidant mechanism of MSRA, inducing an increase in enzyme activity. Another study demonstrated that supplementation with water containing the GSH precursor N-acetyl-L-cysteine (NAC) in mice that express human *α*-synuclein promotes a decrease in *α*-synuclein in the brain and protects, at least partially, the decrease in dopamine concentrations, characteristics that were associated with the reduction in nuclear factor kappa B (NF-*ĸ*B) [195].

*(3) Hydrogen Sulfide*. Hydrogen sulfide is a gaseous neurotransmitter with neuroprotective effects. Sarukhani et al. [196] investigated its activity in an acute 6-OHDA animal model of PA and concluded that hydrogen sulfide produces a significant antiparkinsonism effect, protecting against 6-OHDA neurotoxicity, as it reduces malondialdehyde overproduction.

*(4) N-Acetylcysteine*. Known as an antioxidant and a GSH synthesis precursor for a long time, NAC was studied in two independent studies with similar results. Virel et al. [197], working with human mesenchymal cells, and Bonilla-Porras et al. [198], working with mice, demonstrated that 6-OHDA treatment caused GSH depletion that was not reversed by NAC cotreatment, despite the fact that this treatment improved antioxidant levels in both studies. According to the authors, this highlights the importance of GSH on brain metabolism. Moreover, Coles et al. [199] treated PD patients with repeated oral doses of NAC, but no changes in oxidative stress markers were observed, despite increased levels of antioxidant markers of PD patients in comparison with healthy controls.

### 3.2. Vitamins

The beneficial effects of antioxidant vitamins in PD were evaluated in a series of studies that assessed dietary vitamin intake using structured questionnaires. Indeed, Miyake et al. [200] evaluated the relation between the intake of antioxidant vitamins present in vegetables and fruit and the risk of patients developing PD in Japan, observing that a greater consumption of vitamin E and *β*-carotene is associated with a reduction in the risk of PD in this population. Moreover, Rijk et al. [201], studying 5342 individuals from the Rotterdam Study, suggested that high dietary intake of vitamin E may protect against the occurrence of PD. Similar results were found by Zhang et al. [202], Etminan et al. [203], and Schirinzi et al. [204]. Nevertheless, other authors failed to prove the beneficial effects of dietary antioxidant vitamin intake [133, 205–208], suggesting that the vitamin amount provided by the diet is insufficient [209]. Indeed, a recent cohort study conducted by Hughes et al. [210] investigated the intake of vitamins C and E and carotenoids on the risk of PD development and concluded that there are still no results that support the hypothesis that ingestion, alone or a combination of these antioxidant substances, decreases the risk of developing PD. Notwithstanding, in an experimental study, vitamin A and *β*-carotene dose-dependently destabilized preformed *α*-synuclein filaments [211], and the treatment of PD patients with *α*-tocopherol and ascorbic acid delays disease progression [212]. Moreover, research using 6-aminolevulinic acid in an experimental model of PD demonstrated the neuroprotective action of vitamin E through behavioral and histochemical evidence [213]. Zhu [132] suggests that in addition to vitamin C, other antioxidants are important in the diet for the reduction of the risk of PD, such as vitamins B6 and B12, S-adenosyl-L-methionine (SAME), and folic acid, based on the regulation of catechol-O-methyltransferase (COMT), an enzyme that acts in catecholamine degradation.

### 3.3. Phenols and Polyphenols

#### 3.3.1. Tyrosol

A simple phenol present in extra virgin oil, tyrosol, was demonstrated to delay *α*-synuclein aggregation in a *Caenorhabditis elegans* model of PD. Additionally, tyrosol treatment reduced ROS levels and promoted the expression of specific chaperones and antioxidant enzymes [214].

#### 3.3.2. Tricetin

Extracted from *Ginkgo biloba*, tricetin was demonstrated to confer neuroprotection against 6-OHDA-induced oxidative stress in a *C. elegans* model of PD. Moreover, it also induced the protein expression of Nrf2 and its transcriptional activation, resulting in the upregulated expression of heme oxidase-1 [215].

#### 3.3.3. Chrysin

Chrysin is a natural flavonoid found in bee propolis, honey, and several plants and was investigated in both the 6-OHDA [216] and the MPTP [217] models of PD, reversing neurochemical deficits, behavioral abnormalities, and oxidative stress in those animals.

#### 3.3.4. Acteoside

Acteoside is a flavonoid reported to have antioxidant and neuroprotective effects. Li et al. [218] investigated its effect in a 6-OHDA zebrafish model of PD, demonstrating its ability to reduce neural damage and even prevent neural damage. In addition, pretreatment with acteoside could upregulate antioxidant enzymes by activating the Nrf2 signaling pathway.

#### 3.3.5. Pinostrobin

Another flavonoid with antioxidant effects, pinostrobin was also used in the MPTP zebrafish model of PD with similar results as acteoside, as it significantly enhances Nrf2 expression and upregulates heme oxygenase-1 (HO-1) expression [219].

#### 3.3.6. Curcumin

Like other flavonoids, curcumin is reported to have antioxidant and neuroprotective properties. Indeed, its use in both *Drosophila melanogaster* and 6-OHDA-induced PD in rat models resulted in improved locomotive abilities, less severe neurodegeneration, and decreased oxidative stress markers [220–221]. Similar results were found with demethoxycurcumin, a curcumin derivative [222].

#### 3.3.7. Hesperidin

Hesperidin was reported to reduce the iron content in the heads of *D. melanogaster* and to restore dopamine levels and cholinergic activity, as well as to reduce Fe-induced mortality, oxidative stress, and mitochondrial dysfunction in this model of PD [223].

#### 3.3.8. Naringenin

A citrus fruit flavanone, naringenin was employed in two independent studies using the MPTP-induced PD model in mice, leading to an overall reversion of PD-induced features, such as *α*-synuclein aggregation, as well as to lower oxidative stress levels and increased antioxidant parameters [224–225].

#### 3.3.9. Resveratrol

Resveratrol is a very promising polyphenol, showing inhibition of *α*-synuclein aggregation in PD-induced mice [226], increased lifespan of MPTP-treated *D. melanogaster* [227], and protection for PC12 cells from rotenone oxidative damage, an effect partially mediated through the activation of the SIR/Akt1 signaling pathway [228]. In all three studies, oxidative stress was decreased in the resveratrol-treated groups, whereas antioxidant status was increased.

#### 3.3.10. Genistein

Wu et al. [229] investigated the effects of genistein against the rotenone-induced PD model in human SH-SY5Y cells, which express a mutant form of *α*-synuclein. The authors demonstrated that genistein was able to prevent mitochondrial oxidative damage caused by rotenone to those cells. Further investigation led the authors to conclude that genistein can reduce oxidative stress damage and cell apoptosis, activating estrogen receptors and NF-E2L2 channels.

#### 3.3.11. Rosmarinic Acid

Qu et al. [230] demonstrated that rosmarinic acid protected against iron-induced *α*-synuclein aggregation by upregulating HO-1 and inhibiting *α*-synuclein expression.

#### 3.3.12. Salidroside

Wu et al. [231] administered salidroside to 6-OHDA-induced PD rats and demonstrated neuroprotection against oxidative stress, an effect probably related to the regulation of the Wn/*β*-catenin signaling pathway.

#### 3.3.13. Anacardic Acids

Anacardic acids are alkyl phenols mainly present in cashew nuts and were used to treat rotenone-induced PD in rats. Among several of the beneficial effects, the authors demonstrated that the use of anacardic acids prevented motor impairment and lipoperoxidation induced by rotenone, in part due to a modulatory action on mitochondria and SOD gene expression [232].

### 3.4. Terpenes

#### 3.4.1. Thymol

Thymol is a dietary monoterpene and was tested to prevent neurotoxicity and neurodegeneration in rotenone-challenged rats. Rotenone-induced neurodegeneration is a well-established PD model with oxidative stress involvement that mimics the features of PD in humans. Thymol treatment significantly reduced dopaminergic neural loss and oxidative stress, resulting in clinical improvement to the animals and preservation of antioxidant defenses, as well as attenuation of inflammatory mediators [233].

#### 3.4.2. Astragaloside IV

A triterpene extracted from the roots of *Astragalus membranaceus*, an herb known as *Huang Qi* that has been used for more than 5000 years in China, possesses anti-inflammatory and antioxidant properties in neurogenerative diseases and was employed to prevent damage caused by MPTP in rats and lipopolysaccharide-induced damage to BV2 microglial cells with promising results [234].

#### 3.4.3. Carvacrol

It is a phenolic monoterpenoid that is found primarily in the essential oil from oregano. Haddadi et al. [235] treated 6-OHDA-induced PD in rats with carvacrol, showing that a dose of 25 mg promoted significant memory deficit improvement in the animals.

#### 3.4.4. *β*-Amirin

This pentacyclic triterpenoid compound is found in several medicinal plants and promotes excellent antioxidant activity, significantly reducing ROS in a *C. elegans* model of PD. Moreover, *β*-amirin treatment also exerted a protective effect on dopaminergic neurons, reducing cell damage and *α*-synuclein aggregation [236].

#### 3.4.5. Asiatic Acid (AA)

A triterpenoid used for the treatment of depression, asiatic acid is known for its antioxidant properties. AA was tested in three different PD models: PD transgenic *Drosophila* flies, where it caused significant improvement in climbing ability and prolonged the lifespan—effects attributed to AA antioxidant properties; rotenone-induced damage in SH-SY5Y cells, where it protected mitochondria from oxidative stress and apoptosis; and in an isolated mitochondria model, where AA promoted membrane integrity and ATP production against the decline in membrane potential induced by *α*-synuclein. Considering that maintaining mitochondrial integrity is essential in PD, the authors suggested AA as an excellent candidate for PD prevention and therapy [237].

#### 3.4.6. Geraniol

An acyclic monoterpene found in the essential oils of several aromatic plants, geraniol was used to prevent rotenone-induced mitochondrial damage in SK-N-SH cells, ameliorating intracellular redox status, preserving membrane potential, and reducing the expression of *α*-synuclein, features that corroborate enhanced cell viability [238].

### 3.5. Plant Extracts

Beyond using purified antioxidant molecules, several studies have considered the use of crude plant extracts to treat PD-like symptoms and the consequent morphological and biochemical modifications induced in PD models, mainly due to the synergistic effect of the antioxidant molecule content of such extracts. Some of these studies are summarized below.

#### 3.5.1. Grape Skin

Moderate red wine consumption is considered to confer several health benefits, including protection against neurological diseases. These health benefits are suggested to come from resveratrol, a compound from grape skin that displays anti-PD effects [226–228]. Notwithstanding, Wu et al. [239] investigated the effects of grape skin extract (GSE) left from red wine production on a *Drosophila* model of PD, resulting in preservation of mitochondrial morphology and improvement of indirect flight muscle function, as well as in prolonged lifespan of the flies. Notably, the authors suggested that these effects of GSE are not accounted for by resveratrol alone.

#### 3.5.2. *Centella asiatica*

It is a well-known medicinal plant native to southern Asia, Australia, and some Pacific Islands commonly used against circulatory dysfunction in Chinese traditional medicine. Teerapattarakan et al. [240] used a *C. asiatica* extract to treat rotenone-induced PD in rats and showed significant improvement in the travelled distance of treated rats, alongside a higher number of dopaminergic neurons in the *substantia nigra* and *striatum*, decreased MDA, and increased SOD and catalase expression.

#### 3.5.3. *Dendropanax morbifenus*

This plant is an endemic species of South Korea that is extensively used in traditional medicine to treat several clinical complications. Park et al. [241] successfully used *D. morbifenus* leaf extracts to prevent behavioral deficits and dopaminergic neuron loss in the MPTP-induced PD mouse model. Chromatographic profiling of the extract identified chlorogenic acid as its major constituent, a well-known antioxidant agent.

#### 3.5.4. *Azadirachta indica*

Similar to *D. morbifenus*, *A. indica* is a medicinal plant used for more than 2,000 years in India and displays anti-PD properties. Curiously, it is called “*arishtha*” in Sanskrit, which means “the eliminator of pain.” Indeed, treatment with *A. indica* extract to 6-OHDA-induced PD rats promoted improved motor behavior and reversed several biochemical modifications induced by 6-OHDA, such as the suppression of inflammatory factors, antioxidant enzymes, and iNOS expression [242].

#### 3.5.5. *Zizyphus spinachristi*

Known as “Christ's thorn jujube,” *Zizyphus spinachristi* is an evergreen tree native to northern and tropical Africa and Southern and Western Asia. Fruits and leaves from the tree have been used in Ancient Egypt as food and medicine. Singh et al. [243] investigated the beneficial effects of *Z. spinachristi* fruit extract against MPTP-induced neurotoxicity in SH-SY5Y cells, demonstrating its ability to reverse cell damage and oxidative stress, effects accounting for its potent antioxidant properties.

#### 3.5.6. *Apium graveolens* L.

*Apium graveolens L.* is used in Chinese traditional medicine and is routinely prescribed for the treatment of gout, diabetes, and hypertension. Chonpathompikunlert et al. [244] tested the effect of the methanolic extract of the whole plant on the MPTP model of PD in rats and demonstrated significant improvement in behavioral performance and oxidative stress parameters, as well as an increased number of dopaminergic neurons.

#### 3.5.7. *Ginkgo biloba*

Another potent antioxidant tested was the extract rich in flavonoids and terpenes obtained from leaves of *Ginkgo biloba*, which promoted effective protection to the neurons of animals exposed to MPTP in an experimental model of PD [245–246].

#### 3.5.8. *Aspidosperma pyrifolium* Mart.

*Aspidosperma* species are commonly used in folk medicine in Brazil, especially to treat malaria, and there are several ongoing studies in this regard. Among them, *A. pyrifolium* Mart. aqueous extract was tested against 6-OHDA-induced PD in rats, where the treated groups showed decreased PD features, including less lipid peroxidation and increased levels of dopamine, suggesting a potential for this extract in PD treatment [247].

#### 3.5.9. *Olea europaea* L.

Leaf extract from this ordinary olive tree has shown antioxidant and neuroprotective effects, which led Sarbishegi et al. [248] to investigate its effect against a rotenone-induced model of PD in rats, resulting in significant improvement of oxidative markers and blockage of depletion of tyrosine hydroxylase-positive neurons caused by rotenone exposure.

#### 3.5.10. *Bacopa monnieri*

This plant is used in Ayurvedic medicine for the treatment of neurological disorders and displays high levels of antioxidant molecules. Tested against the MPTP-induced PD model in mice, the ethanolic extract of *B. monnieri* treatment promoted several anti-PD effects, including modulation of oxidative stress and nigrostriatal dopaminergic neuroprotection [249].

#### 3.5.11. *Hibiscus asper*

The methanolic extract of the leaves of *Hibiscus asper* was used in an experimental model of PD in rats and proved to be neuroprotective, as it provided a significant increase in the activity of antioxidant enzymes (SOD, CAT, and GSH-Px) and decreased lipid peroxidation in the brain [250].

#### 3.5.12. Blackberries

The ethanolic extract of blackberries was used in both *in vitro* and *in vivo* models of PD and demonstrated dose-dependent neuroprotective effects through antiapoptotic and antioxidant effects [251].

#### 3.5.13. *Eplingiella fruticosa*

*Eplingiella fruticosa* is a Brazilian aromatic plant used for pain treatment in Brazilian folk medicine, which has demonstrated potent antioxidant and anti-inflammatory properties. Beserra-Filho et al. [252] tested the essential oil obtained from *E. fruticosa* leaves against a reserpine-induced PD model in mice and demonstrated important anti-PD effects, such as modulation of oxidative stress, delayed onset of catalepsy, and protection against dopaminergic depletion in the *striatum*.

#### 3.5.14. Red Ginseng

Ginseng treatment of rotenone-induced PD in rats promoted marked improvement of locomotor activity, suppression of *β*-amyloid deposition, and inhibition of the NF-*ĸ*B inflammatory pathway and oxidative stress mediators, and significantly increased tyrosine hydroxylase activity [253]. Moreover, *Angelica sinensis* extract, popularly known as “female ginseng,” also prevented the occurrence of PD-like symptoms in a *C. elegans* model of the disease [254].

#### 3.5.15. Seaweeds

Using the 6-OHDA-induced PD model in SH-SY5Y human neuroblastoma cells, several studies demonstrated promising anti-PD effects of seaweed extracts, such as brown seaweeds *Bifurcaria bifurcata* [255], *Ecklonia cava* [256], and *Sargassum hemiphyllum* [257], as well as the red seaweed *Chondrus crispus*, which was tested on the *C. elegans* model of PD [258].

### 3.6. Other Plant-Derived Molecules

#### 3.6.1. Diosgenin

This natural steroid saponin extracted from the tubers of *Dioscorea* wild yam was used to prevent the alterations caused in the lipopolysaccharide- (LPS-) induced PD model in rats, resulting in a significant reduction in oxidative stress markers and inactivation of the Toll-like receptor (TLR)/NF-*ĸ*B inflammatory pathway [259].

#### 3.6.2. Thymoquinone

Extracted from the seeds of *Nigella sativa*, a plant popularly known as black cumin, thymoquinone is a bicyclic benzenoid ketone. It was employed by Ardah et al. [260] to prevent MPTP-induced PD in mice. Treatment with thymoquinone restored antioxidant enzymes, prevented lipid peroxidation, and attenuated the expression of proinflammatory cytokines.

#### 3.6.3. Sulforaphane

It is an organic isothiocyanate extracted from many cruciferous vegetables, such as cabbages and broccolis. Bao et al. [261] investigated its effect on MPTP-induced damage in PC12 cells, reporting its ability to reduce Nrf2, HO-1, and nicotinamide quinine oxidoreductase, concluding that sulforaphane protected the cells via activation of the Nrf2-antioxidant responsive element pathway.

#### 3.6.4. Crocin

Crocin, a saffron-active component, exhibited protective effects against malathion-induced PD in rats by reducing oxidative stress and anti-inflammatory effects and improving motor deficits and neurobehavioral impairments [262].

#### 3.6.5. Spermidine

Spermidine is an antioxidant polyamine and was tested against rotenone-induced PD in rats, reversing neuroinflammation and restoring striatal neurochemistry, as well as oxidative stress markers [263].

#### 3.6.6. Gastrodin

It is the glucoside of gastrodigenin and has been isolated from the orchid *Gastrodia elata*. Haddadi et al. [264], using the 6-OHDA model of PD in rats, demonstrated catalepsy prevention and motor coordination in lesioned rats. Moreover, gastrodin suppressed MPO activity, lipid peroxidation, and NO synthesis induced by 6-OHDA and increased total antioxidant capacity in the *substantia nigra pars compacta* of these rats.

### 3.7. Drugs

#### 3.7.1. Paroxetine

Using a mouse experimental model, Chung et al. [123] studied the effects of the antioxidant paroxetine in mice that received MPTP, demonstrating that this antidepressant drug protects *nigrostriatal* dopaminergic neurons from oxidative damage induced by the neurotoxin. Additionally, the authors also verified that paroxetine inhibited microglial activation and, therefore, the expression of iNOS and TNF-*α*; inhibited the activation of *astroglia* and hence the production of MPO; and promoted attenuation of the production of oxidizing agents via NADPH oxidase. Collectively, oxidative stress reduction has enabled the increase of dopamine levels in the *nucleus striatum* and the improvement of the motor performance of these animals.

#### 3.7.2. Pramipexole

Pramipexole is a novel dopamine agonist that also inhibits oxidative stress and mitochondrial apoptosis. Wang et al. [265] used pramipexole transdermal patches (PPX) against MPTP-induced PD in mice, showing that PPX improved dyskinesia in PD-induced mice and restored the activity of antioxidant enzymes alongside MDA reduction. Another similar study demonstrated that PPX activates Akt kinase and, therefore, is related to SPHK1 activation, which is crucial for neurite extension in neurons and directed cell movement [266].

#### 3.7.3. Simvastatin

Regularly employed to reduce cholesterol levels, this hydroxy-methyl-glutaryl-coenzyme A reductase inhibitor was tested against the 6-OHDA model of PD both in SH-SY5Y cells and mice, causing a reduction in oxidative markers, reversion of apoptosis, and inhibition of the mitogen-activated protein kinase (MAPK) pathway and NF-*ĸ*B activation in SH-SY5Y cells. Simvastatin treatment in mice decreased limb asymmetry and apomorphine-induced rotations in PD mice [267].

#### 3.7.4. Methylene Blue

Clinically used for a relatively long time, methylene blue is known for its neuroprotective and antioxidant properties. Focusing on the induction of neurotrophic factors, Bhurtel et al. [268] studied its effects against MPTP-induced PD in both *in vivo* and *in vitro* models of the disease. According to the authors, methylene blue treatment significantly reduced the loss of dopaminergic neurons, depletion of dopamine, and glial cell activation through the activation of brain-derived neurotrophic factor (BDNF).

#### 3.7.5. Ebselen

MPTP was also used in a study performed in the primate model of PD to observe the action of ebselen, an antioxidant with actions similar to glutathione peroxidase. It was demonstrated that ebselen could prevent both the loss of neurons and the onset of clinical symptoms of the disease in this experimental model [269].

#### 3.7.6. Geranylgeranylacetone

This synthetic drug used to treat gastric ulcers was associated with glial cell-derived neurotrophic factor against the MPTP-induced PD model in mice. Treated animals displayed significant recovery in their swim, pole, and traction scores, as well as reduced neuronal apoptosis in the *substantia nigra* and oxidative stress markers [270].

#### 3.7.7. Lactoferrin

It is a non-heme iron-binding glycoprotein belonging to the transferrin family and was tested against the MPTP model of PD in mice. Beneficial effects on both the central and peripheral systems were observed, including a reduction in oxidative stress and neuronal apoptosis [51].

#### 3.7.8. Apocynin

This well-known NADPH oxidase inhibitor was used by Hou et al. [271] to treat mice induced to PD by pesticide exposure (paraquat and maneb), causing significant improvement of mouse learning and memory deficits, effects associated with the inhibition of signal transducers and activators of transcription 1 (STAT1) and NF-*ĸ*B pathways.

#### 3.7.9. Norfluoxetine

Norfluoxetine is an active metabolite of the antidepressant fluoxetine that inhibits serotonin uptake. Treatment with norfluoxetine inhibited NADPH oxidase activation and nitrate production in microglial cell cultures and mitigated microglial cell activation and microglial-derived ROS production in the MPTP model of PD in mice [272].

#### 3.7.10. Phenothiazine

It was formerly used as an insecticide and as a drug to treat infections with parasitic worms (anthelminthic) in livestock and humans and is the mother drug of modern antipsychotic drugs. Tapias et al. [273] used it against the rotenone-induced PD model in rats and rat midbrain cell cultures, demonstrating a significant reduction in protein thiol oxidation, mitochondrial dysfunction, axonal impairment, oxidative stress, and inflammatory response as a result of phenothiazine treatment.

#### 3.7.11. Hydralazine

A potent Nrf2 activator, hydralazine was used by Guo et al. [274] against the MPTP-induced PD model in SH-SY5Y cells and mice, resulting in significant translocation of Nrf2, as well as upregulation of the expression of its downstream antioxidant genes. These effects resulted in substantial improvements in oxidative stress, behavioral disorders, and the loss of dopaminergic neurons in the *substantia nigra* and striatum of treated mice and cells, effects attributed to Nrf2 pathway activation.

### 3.8. Other Synthetic Molecules

#### 3.8.1. Montelukast (MK)

A cysteinyl leukotriene receptor antagonist, MK later exhibited remarkable neuroprotective activity in various neurodegenerative disorders. In the rotenone-induced PD animal model, MK exhibited neuroprotective effects through the attenuation of microglial cell activation, oxidative stress inhibition, and p38 MAPK expression [275].

#### 3.8.2. DDO-7263

A novel Nrf2-ARE activator, DDO-7263 was tested against MPTP-induced PD in mice, improving behavioral abnormalities induced by MPTP and significantly attenuating chemically induced dopaminergic neuron loss of tyrosine hydroxylase in the *substantia nigra* and *striatum*. In addition, DDO-7263 inhibited the secretion of inflammatory factors and protected PC12 neurons from H_2_O_2_-induced oxidative damage [276].

#### 3.8.3. KMS99220

A synthetic morpholine-containing chalcone, KMS99220 confers neuroprotection due to its high binding affinity to the Nrf2 inhibitory protein Keap-1 and increased nuclear translocation of Nrf2 and gene expression of the antioxidant enzymes. It is reported to reduce *α*-synuclein aggregates in GFP-*α*-syn A53T-overexpressing cells, and in MPTP-treated mice, oral administration of KMS99220 prevented degeneration of the nigral dopaminergic neurons, induced the Nrf2 target genes, and effectively prevented the associated motor deficits [277].

#### 3.8.4. M40403

A SOD-mimetic compound, it was employed with positive results in cellular and fly models of PD to reverse PD symptoms in PINK1 and Parkin phenotypes, which are known to be associated with early-onset forms of PD [278].

### 3.9. Use of Nanoparticles to Deliver Antioxidants

Another interesting method of antioxidant treatment in PD consists of delivering antioxidant molecules through nanoparticles that can direct antioxidant effects towards specific sites of the cell or that display specific scavenging activities.

#### 3.9.1. Ceria Nanoparticles

Ceria nanoparticles effectively scavenge ROS, present catalase- and SOD-mimetic activities, and readily penetrate the cellular membrane and scavenge intracellular ROS in the cytosol. Moreover, triphenylphosphonium-conjugated ceria nanoparticles can scavenge mitochondrial ROS after their delivery to mitochondria. Extracellular ROS can also be scavenged through 300 nm sized ceria nanoparticle clusters that are not subject to cellular uptake. Kwon et al. [279] used ceria nanoparticles to treat PD-like symptoms in the MPTP model of PD in mice, reporting inhibition of lipid peroxidation and protection of tyrosine hydroxylase in the *striatum* of treated mice.

#### 3.9.2. Chitosan Nanoparticles

Raj et al. [280] used chitosan nanoparticles to deliver the PD drug pramipexole by the intranasal route to rotenone-induced PD rats, reporting enhanced antioxidant status and increased dopamine levels in treated animals.

#### 3.9.3. Nanoemulsions

Nanoemulsions were also tested to deliver selegine (also known as L-deprenyl, a medication that is used in the treatment of PD), displaying high antioxidant properties that, along with the anti-PD effects of selegine, conferred significant protection to treated rats [281].

## 4. Final Remarks

The use of antioxidants as adjuvant PD therapy has been debated because the results of the efficacy of antioxidant substances are not yet fully clarified in human studies. Nevertheless, although there is no clarity regarding the efficacy of antioxidant use in PD patients, Agim and Cannon [282] point out that dietary components may act as protective factors in PD, as demonstrated in both *in vivo* and *in vitro* studies.

Indeed, as presented in the numerous reports cited in this review, *in vitro* studies and animal models provide vast and strong evidence for the benefits of antioxidant supplementation to treat PD and set a solid ground for its use in human studies.

Among the several antioxidant approaches reported, antioxidants derived from plants have presented remarkable results, especially those with high flavonoid content, such as purple and red fruits and seaweeds.

In this sense, Joseph et al. [283] believe that antioxidant-rich foods may benefit neurons during neuronal aging, reversing or delaying free radical action, which are normally produced by dopaminergic neurons of the *substantia nigra* of the brain.

Nevertheless, it is worth noting that free radicals exert several beneficial roles in mammalian cells, such as ATP production, phagocytosis, and cell signaling [82], and the indiscriminate use of antioxidants might be harmful.

In conclusion, oxidative stress plays a crucial role in the pathogenesis of Parkinson's disease, either by external factors or individual intrinsic factors. Nevertheless, the effects of oxidative stress and other factors related to the disease have not been fully elucidated thus far, and further studies are still necessary in the search for the formulation of new drugs and for more efficient use of existing drugs. However, the potential benefit of antioxidant supplements as an adjuvant therapy for Parkinson's disease is unquestionable and is aimed at improving patient quality of life.

## Abbreviations

6-OHDA: 6-Hydroxydopamine
8-OHdG: 8-Hydroxy-2-deoxyguanosine
AA: Asiatic acid
BDNF: Brain-derived neurotrophic factor
BH4: Tetrahydrobiopterin
CAT: Catalase
Cl: Chloride ion
COMT: Catechol-O-methyltransferase
CoQ_10_: Coenzyme Q_10_
COX-2: Cyclooxygenase-2
DHA: Docosahexanoic acid
DHLA: Dihydrolipoic acid
DOPAC: Dihydroxyphenylacetic acid
EP: Ethyl pyruvate
Fe^2+^: Ferrous ion
G6PD: Glucose-6-phosphate dehydrogenase
GFAP: Glial fibrillary acidic protein
GR: Glutathione reductase
GRP75: Glucose-regulated protein 75
GSE: Grape skin extract
GSH: Reduced glutathione
GSH-Px: Glutathione peroxidase
GSSG: Oxidized glutathione
GST: Glutathione-S-transferase
H_2_O_2_: Hydrogen peroxide
HO-1: Heme oxygenase-1
HOC: Hypochlorous acid
IL-1*β*: Interleukin 1 beta
iNOS: Inducible nitric oxide synthase
KA: Kynurenic acid
LA: Lipoic acid
L-DOPA: L-Dihydroxyphenylalanine
LOOH: Lipid hydroperoxides
LPS: Lipopolysaccharide
MAO: Monoamine oxidase
MAPK: Mitogen-activated protein kinase
MDA: Malondialdehyde
MK: Montelukast
MPDP^+^: Dihydropyridine
MPO: Myeloperoxidase
MPP^+^: N-Methyl-4-phenylpyridine
MPTP: 1-Methyl-4-phenyl-1,2,3,6-tetrahydropyridine
MSRA: Methionine sulfoxide reductase
mtHsp70: Mitochondrial heat-shock protein 70
*n*-3 PUFA: Omega-3-polyunsaturated fatty acids
NAC: N-Acetylcysteine
NAD: *β*-Nicotinamide adenine dinucleotide
NADPH oxidase: Reduced nicotinamide adenine dinucleotide phosphate oxidase
NF-E2: Factor nuclear erythroid 2
NF-*κ*B: Nuclear factor kappa *β*
nNOS: Neuronal nitric oxide synthase
NO: Nitric oxide
NO_2_: Nitrite
NO_2_^•^: Free radical form of nitrite
NOS: Nitric oxide synthase
NOx: Nitrites and nitrates
NQO1: Quinone oxidoreductase 1
Nrf2-ARE: NF-E2-related factor 2 bound to the antioxidant response element pathway
Nrf2: NF-E2-related factor 2
O_2_^•^: Superoxide
OH^•^: Hydroxyl radical
ONOO^:^: Peroxynitrite radical
PD: Parkinson's disease
PINK1: PTEN-induced putative kinase 1
PPX: Pramipexole transdermal patches
QA: Quinolinic acid
RNS: Reactive nitrogen species
ROS: Reactive oxygen species.
SAME: S-Adenosyl-L-methionine
SOD: Superoxide dismutase
STAT1: Signal transducers and activators of transcription 1
TH: Tyrosine hydroxylase
TLR: Toll-like receptor
TNF-*α*: Tumor necrosis factor alpha.
